# Transcriptomic analysis reveals mode of action of butyric acid supplementation in an intensified CHO cell fed‐batch process

**DOI:** 10.1002/bit.28150

**Published:** 2022-06-24

**Authors:** Markus Schulze, Yadhu Kumar, Merle Rattay, Julia Niemann, Rene H. Wijffels, Dirk E. Martens

**Affiliations:** ^1^ Product Development Cell Culture Technologies Sartorius Stedim Biotech GmbH Göttingen Germany; ^2^ Bioprocess Engineering Wageningen University Wageningen The Netherlands; ^3^ Eurofins Genomics Europe Sequencing GmbH Konstanz Germany; ^4^ Corporate Research Advanced Cell Biology Sartorius Stedim Cellca GmbH Ulm Germany; ^5^ Corporate Research BioProcessing Upstream Sartorius Stedim Biotech GmbH Göttingen Germany; ^6^ Biosciences and Aquaculture Nord University Bodø Norway

**Keywords:** butyric acid, CHO cell culture, intensified fed‐batch, N‐1 perfusion, process intensification, transcriptomics

## Abstract

Process intensification is increasingly used in the mammalian biomanufacturing industry. The key driver of this trend is the need for more efficient and flexible production strategies to cope with the increased demand for biotherapeutics predicted in the next years. Therefore, such intensified production strategies should be designed, established, and characterized. We established a CHO cell process consisting of an intensified fed‐batch (iFB), which is inoculated by an N‐1 perfusion process that reaches high cell concentrations (100 × 10^6^ c ml^−1^). We investigated the impact of butyric acid (BA) supplementation in this iFB process. Most prominently, higher cellular productivities of more than 33% were achieved, thus 3.5 g L^−1^ of immunoglobulin G (IgG) was produced in 6.5 days. Impacts on critical product quality attributes were small. To understand the biological mechanisms of BA in the iFB process, we performed a detailed transcriptomic analysis. Affected gene sets reflected concurrent inhibition of cell proliferation and impact on histone modification. These translate into subsequently enhanced mechanisms of protein biosynthesis: enriched regulation of transcription, messenger RNA processing and transport, ribosomal translation, and cellular trafficking of IgG intermediates. Furthermore, we identified mutual tackling points for optimization by gene engineering. The presented strategy can contribute to meet future requirements in the continuously demanding field of biotherapeutics production.

## 1 INTRODUCTION

Mammalian expression systems are the workhorse of biotherapeutics production among which Chinese hamster ovary (CHO) cells are the predominantly used production platform (O'Flaherty et al., 2020). CHO cells are well characterized and yield reasonable productivities and favorable glycosylation patterns of complex proteins (Tejwani et al., 2018), such as monoclonal antibodies (mAb). The dynamically growing market of all these therapeutics currently compiles to an estimated value of US$ 237.2 billion (Lu et al., 2020). In the context of the SARS‐CoV‐2 pandemic, the need for rapid development of effective therapeutics and a scalable and productive process strategy has become apparent in the recent past (Weinreich et al., 2021). Additionally, biosimilars will enter the markets as former blockbuster drugs are running off patent. Thus, both the demand and cost pressure (Farid et al., 2020; Paul et al., 2010) urge companies to improve the production processes of mammalian biotherapeutics to further participate in the globally expanding market. Process intensification can be one important part of the solution, which is a key trend involving technologies and process strategies to improve productivity and plant utilization by increasing flexibility.

A central cornerstone of process intensification is the use of perfusion processes in mammalian cell cultivation. Cells are retained inside the bioreactor via a cell retention device and spent media is continuously replenished creating optimal steady‐state conditions. This allows to work up to very high cell concentrations and achieve very high volumetric productivities. During the last decade, perfusion processes gained attention and have been increasingly studied (MacDonald et al., 2021). A relevant technology to control perfusion processes is the application of suitable online process analytical technology. This technology helps to retain and cultivate (up to or at) a desired number of cells under optimal steady‐state conditions with respect to their physiological and nutritional needs. Perfusion at the N‐1 stage in a cell culture process allows to attain very high viable cell concentrations (VCC). These can be used to increase the inoculation concentration of the subsequent production process (N‐stage), which is called intensified fed‐batch (iFB). In previous studies, we have implemented N‐1 perfusion with online control of the cell‐specific perfusion rate (CSPR) (Schulze et al., 2021) and used this process to intensify the N‐stage process in small‐scale bioreactors (15 ml) accordingly. By supplementing butyric acid (BA) to an iFB in 250 ml bioreactors, we next substantially improved the average volumetric immunoglobulin G (IgG) productivity by 96% (Schulze et al., 2022). The supplementation of short‐chain fatty acids (SCFA) as a productivity enhancer in conventional mammalian upstream processes is not new, but rather unexplored in intensified processes. Also, the cellular mechanisms influenced by BA are not fully unraveled yet (Jiang & Sharfstein, 2008). In this study, we transfer the process to a bench‐top scale, carefully examine important critical quality attributes, be it the N‐glycosylation profile or charge heterogeneity of the produced IgG, and use transcriptomics to shed light on the underlying mechanisms. In doing so, we evaluate such a process strategy as a viable option to fulfill upcoming demands and requirements. Furthermore, potential tackling points for future process development are identified to alleviate the cytotoxic effects of BA.

## 2 MATERIALS AND METHODS

### 2.1 Seed train using N‐1 perfusion

We used a CHO cell line producing a monoclonal antibody (mAb, IgG1), media platform, and seed train strategy using N‐1 perfusion in 2‐L single‐use bag bioreactors based on rocking motion (BIOSTAT® RM 20| 50, Sartorius) as described in (Schulze et al., 2021). In brief, perfusion was controlled based on online VCC measurements at a CSPR of 50 pL c^−1^ day^−1^, and the cells were cultivated at 36.8°C, 60% DO, and pH 6.95.

### 2.2 N‐stage process intensification

We conducted two different intensified fed‐batches in triplicate in 5‐L stirred benchtop bioreactors (Univessel® Glass; Sartorius). Both processes were inoculated at 5 × 10^6^ c ml^−1^ at a starting volume of 3.33 L. One process was used as control (iFB) and the other one was supplemented with 2.5 mM BA (iFB + BA) on Day 2.5 based on a 100‐mM BA stock solution in the production medium. The following process conditions were applied: *T* = 36.8°C, pH 7.1 via CO_2_ gassing, and DO 60% using a gassing cascade with stirring at 327 rpm (three‐blade segment impeller). Both cultivations used the same feeding scheme: daily additions of feed A and B, with 4% and 0.4% of the starting volume respectively, starting 12 h postinoculation. Glucose was added (400 g L^−1^ stock) if required to reach a concentration of 5 g L^−1^ from Day 1.5 onwards. Daily samples were taken for offline analytics and processed to generate messenger RNA (mRNA) samples for transcriptomic analysis. Processes were terminated as soon as the viability fell below 70%.

### 2.3 Offline analytics

Cell growth (VCC, viability, and average cell diameter) was measured using a Cedex HiRes Cell Counter (Roche). The pH, pO_2_, pCO_2_, as well as metabolite levels (glucose, lactate, ammonia, and l‐glutamine) and osmolality were measured using a BioProfile® FLEX2 (Nova Biomedical). Cell‐free supernatants (stored at −20°C) were used for product quantification via HPLC SEC and analysis of N‐Glycan profiles using a LabChipGXII Touch 24 (PerkinElmer) as described in (Schulze et al., 2022). This analyzer was also used to measure charge variants of the IgG based on capillary zone electrophoresis (CZE) performing the High pI Charge Variant Assay (PerkinElmer) according to the manufacturer's instructions.

### 2.4 Transcriptomics

Directly after sampling, a sample aliquot was stored on ice and the respective volume for 5 × 10^6^ c ml^−1^ was centrifuged (300 g, 5 min, 4°C) in RNase‐free tubes after the results of cell counting. The cell pellet was lysed in RNA lysis buffer, consisting of 350 µl RLT‐buffer (Qiagen) and 7 µl of 2 M 1,4‐dithiothreitol (Sigma‐Aldrich). The lysed cells were transferred to a new RNase‐free tube and stored at −80°C. Random‐primed complementary DNA libraries were prepared and deep sequenced on Illumina NovaSeq. 6000 platform generating at least 30 million 150 bp paired‐end sequencing reads per sample (Eurofins Genomics). Sequencing reads were mapped to the CHO reference genome (*CriGri‐PICRH‐1.0*: GCF_003668045.3; https://www.ncbi.nlm.nih.gov/assembly/GCF_003668045.3) using STAR aligner v2.7.3 (Dobin et al., 2013) followed by quantification of transcripts using RSEM v1.3.3 (B. Li & Dewey, 2011). All genes with more than 10 reads on average were included, resulting in expression data for 13,312 unique genes. Differential gene expression (DGE) analysis was performed using R/Bioconductor DESeq. 2 package v1.19.3 (Bioconductor Package Maintainer et al., 2021; Love et al., 2014) for three groups being: A (Day 6 N‐1 perfusion, when cells were used for inoculation; *n* = 2), B (time series of iFB: d0, d2.5, d3.5, d4.5, d7.5, d8.5; *n* = 3), and C (time series of iFB with butyric acid addition [iFB+BA]: d0, d2.5, d3.5, d4.5, d6.5; *n* = 3). Raw read counts were normalized using DESeq. 2's variance stabilizing transformation following statistical significance test using negative binomial generalized linear models for each gene to compare the distributions between the conditions generating *p* values for each gene. The final *p* values were corrected by determining false discovery rates (FDR) using Benjamin–Hochberg method (Benjamini & Hochberg, 1995). Using FDR corrected *p* value (adjusted *p* value) ≤ 0.1 as a threshold, genes with mean read counts ≥100 and an absolute fold change (FC) of ≤ −1 and ≥1 were defined to be significantly differentially expressed between the groups.

Using the normalized gene counts generated by DESeq. 2 before DGE analysis as input, gene set enrichment analysis (GSEA) was conducted. CHO gene symbols were converted to human gene symbols using a translation table available from http://chomics.org/PICR.tar.gz (Lin et al., 2020). Genes with redundant CHO or human gene symbols and genes without human gene symbols were removed from the normalized gene count tables. The remaining 11,922 genes were subjected to GSEA using the GSEA package (Subramanian et al., 2005) to identify enriched gene sets between the conditions. Gene counts were transformed into ranks with GSEA's default ranking metric (signal‐to‐noise‐ratio) and mapped to the Gene Ontologies (GO) database (Molecular Signature Database [MSigDB] v7.4; considering all categories, that is, biological processes [BP], cellular compartments [CC], and molecular functions [MF]). Gene sets with a size of 15–500 genes were analyzed and considered as being significantly enriched with an FDR adjusted *q* ≤ 0.05.

## 3 RESULTS AND DISCUSSION

### 3.1 Cell culture

#### 3.1.1 Cultivation results

**Figure 1:**
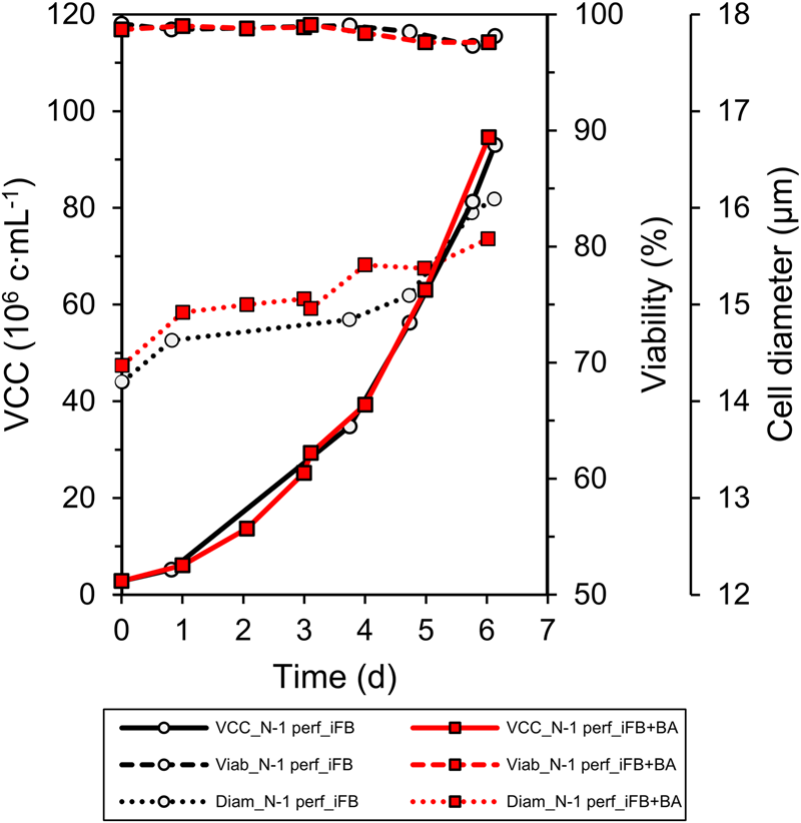
Cell growth characteristics, viability, and cell diameter of the two N‐1 perfusion processes used to grow cells up to almost 100 × 10^6^ c ml^−1^ before inoculation of the iFBs. iFB, intensified fed‐batch; VCC, viable cell concentrations.

A standard iFB and an iFB+BA were conducted in triplicate. Both sets of triplicate runs were inoculated from a separate N‐1 perfusion process. The two N‐1 perfusion processes used were very similar (Figure 1). Both were grown to very high VCCs of almost 100 × 10^6^ c ml^−1^ within 6 days and showed comparable and high viabilities. The average cell diameter at inoculation was comparable at about 16 µm. The consistency in the performance of the N‐1 perfusion process used for inoculation of the two iFB processes is the foundation for subsequent comparisons thereof.

**Figure 2:**
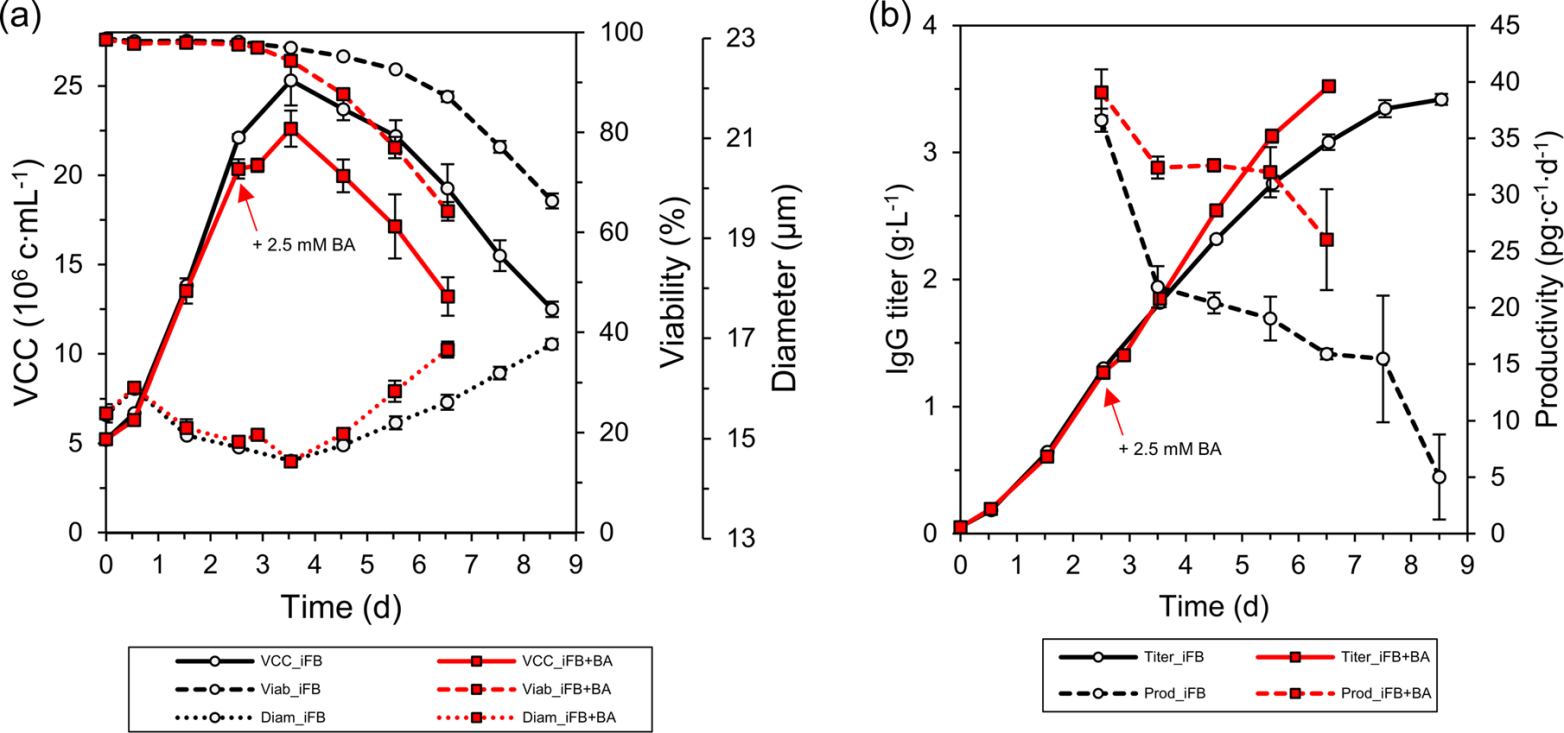
(a) Cell growth characteristics, viability, and cell diameter of the two intensified fed‐batch cultivations. (b) IgG titer and cell‐specific productivity over the course of the processing time. The control (iFB) was cultivated for 8.5 days, while the other process was supplemented with 2.5 mM BA on Day 2.5 (iFB + BA) and cultivated for 6.5 days. iFB, intensified fed‐batch; IgG, immunoglobulin G; VCC, viable cell concentrations.

The two iFB processes were inoculated at 5 × 10^6^ c ml^−1^ in 5 L benchtop bioreactors (Figure 2a). Until day 2.5, they were operated identically and showed comparable growth: Cells grew similarly up to around 20 × 10^6^ c ml^−1^, remained very viable (>98%), and had an equal average cell size (15 µm). On Day 2.5, BA was added to the three experimental runs up to a final concentration of 2.5 mM, while the control runs were left unchanged. This time point and the concentration of BA were identified in a preceding study investigating the impact of different SCFAs on the used cell line in a dose‐dependent manner (Schulze et al., 2022). Peak VCCs for both processes were reached at 3.5 days. While the control reached a peak VCC of 25.3 × 10^6^ c ml^−1^, the process supplemented with BA on Day 2.5 only reached 22.6 × 10^6^ c ml^−1^ due to cell growth reduction. The viability in both processes started to decrease on day 3.5; however, the viability in the butyric acid supplemented cultures Declined faster. The average cell diameter decreased in both processes until Day 3.5 when they reached identical minimal cell diameters (14.7 µm). After Day 3.5, the average cell diameter increased to 17.5 µm with a faster increase for the butyric acid‐treated cultures. Both processes ended as the viability dropped below 70%, which for iFB+BA was on Day 6.5 and for the standard iFB on Day 8.5. The obtained results are very comparable to previous observations made in 250 ml bioreactors (Schulze et al., 2022) with minor differences such as a slightly higher peak VCC (25.3 vs. 21.1 × 10^6^ c ml^−1^) and a lower final diameter (17.5 vs. 18.8 µm).

Both processes reached similar product concentrations of 3.5 g L^−1^ within their process time (Figure 2b), which is in line with previously published results on a 250 ml scale (Schulze et al., 2022). Thus, in the BA‐supplemented iFB product concentrations comparable to the control were achieved in a shorter period of time by consistently higher cell‐specific productivities (*q*_*P*_) after BA supplementation. While the control had to decrease *q*_*P*_ from 20 to only 5 pg c^−1^ day^−1^ at the process end, *q*_*P*_ remained above 25 pg c^−1^day^−1^ for the BA‐supplemented process, thus ensuring a more than 33% higher *q*_*P*_. As BA supplementation improved *q*_*P*_, we also investigated if it had any effect on the product quality.

#### 3.1.2 IgG product quality

**Figure 3:**
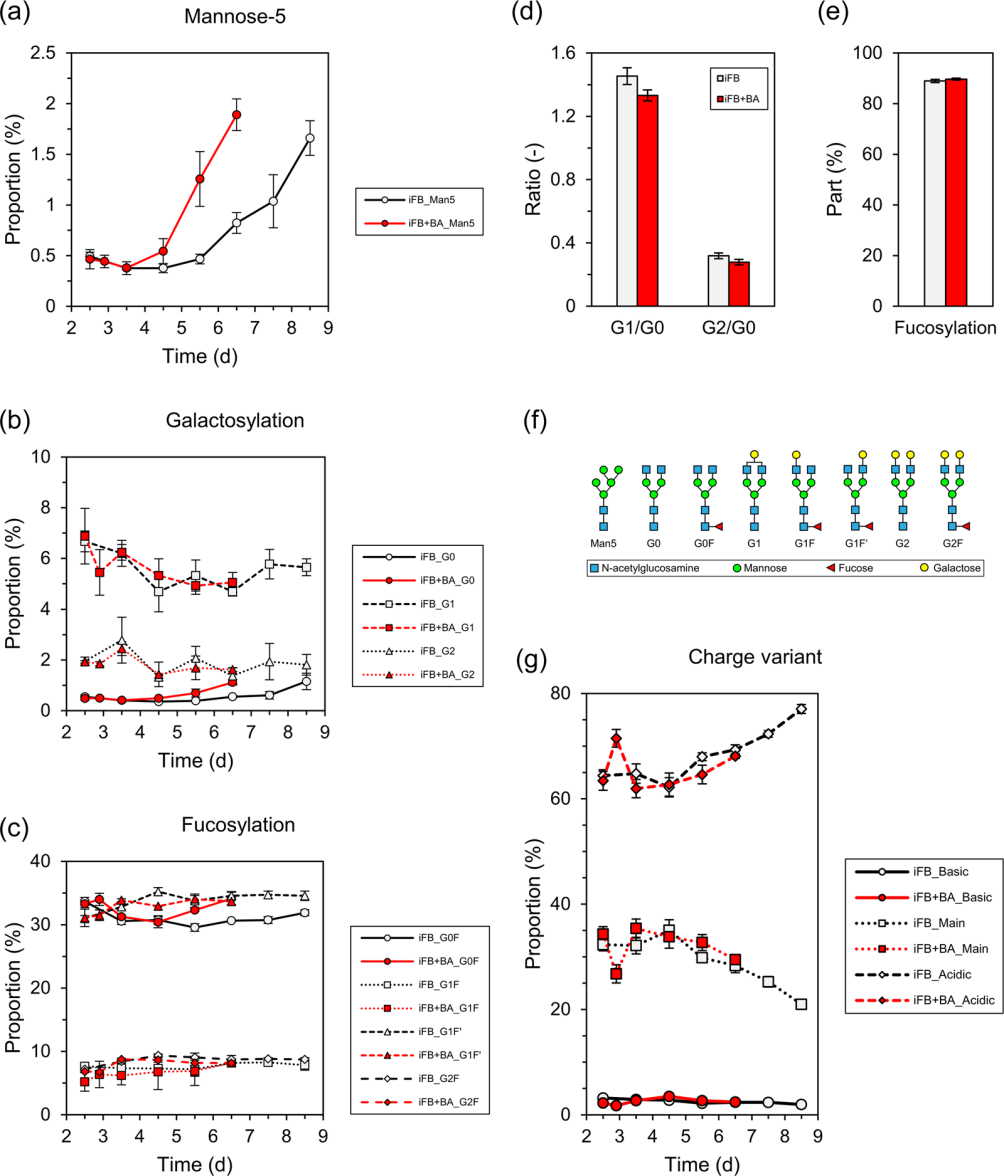
Time profiles of important product quality attributes of the produced IgG for the two different processes iFB and iFB+BA: N‐Glycosylation patterns regarding (a) mannose‐5, (b) galactosylated, and (c) fucosylated isoforms. The final glycan profiles at the end of both processes were compared with respect to (d) galactosylation ratios G1/G0 and G2/G0 as well as (e) total proportion of fucosylation. The analyzed glycan isoforms are schematically represented in (f). The charge variant profile of the IgG is shown in (g). BA, butyric acid; iFB, intensified fed‐batch; IgG, immunoglobulin G.

We measured the product quality (Figure 3) with respect to the N‐glycosylation profile and the charge variant distribution during process time from Day 2.5, when BA was supplemented, until the termination of the process. The proportion of Mannose‐5 (Figure 3a) reached about 1.8% for both processes, which shows an accelerated maturing of IgG for the process iFB+BA. The levels of nonfucosylated glycans (Figure 3b) remained rather constant for both processes and the final composition on Day 6.5 for iFB+BA was very similar to that of Day 8.5 of the iFB, that is, about 5%–6% G1 and 1.5% G2. Only G0 was slightly increased for the process iFB+BA earlier on. The fucosylated glycans (Figure 3c) G1F (8%), G1F' (34%), and G2F (8.5%) were very similar, but G0F was slightly increased in process iFB+BA (34%) compared to iFB (32%). Thus, the total ratio of galactosylated IgG was slightly decreased for iFB+BA (Figure 3d), whereas the proportion of fucosylation (Figure 3e) remained constant at about 89%. These results are consistent with data obtained from an iFB+BA on a 250 ml bioreactor scale (Schulze et al., 2022). With similar final N‐glycosylation profiles in both approaches, it can be assumed that BA does not significantly affect but accelerate the shift/dynamics of identical biological pathways of N‐glycosylation (Sumit et al., 2019) as they occur in the control (iFB) toward the process end. A detailed overview of the process of N‐glycosylation is given in the literature (Fan et al., 2015). Glycosylation starts in the endoplasmic reticulum (ER), where the glycan precursor is built from nucleotide sugars and is attached to the IgG polypeptide chain. After trimming steps catalyzed by glycosidases, the first major type of glycans (high‐mannose) is built. Subsequently, the IgG is translocated into the Golgi apparatus, where the other two major types (hybrid and complex) are created by glycosidases, gycosyl‐ and sialyltransferases. This mechanism consists of several steps, which could be affected by BA supplementation. One option for the reduced galactosylation and accelerated mannosylation is that fewer nucleotide precursors, for example, uridine diphosphate (UDP), are available intracellularly to form building blocks required for the glycosylation procedure, for example, UDP‐galactose (Hills et al., 2001), which may be related to the fact that BA supplementation causes a cell cycle arrest in the G0/G1 phase (Qiu et al., 2017; Schulze et al., 2022). Thus, cells stop growing as shown here (Figure 2) and do not enter the S phase where DNA is replicated and nucleotides must be amply available. Another contributing factor could be elevated osmolality (Supporting Information: Figure S1). It increased following BA supplementation since cells stopped growing, but feeding was the same as in the untreated process. Higher osmolalities have been reported previously to be related to increased mannosylation (Pacis et al., 2011). Furthermore, the increase in extracellular ammonia concentration in the last cultivation days (in both processes, but earlier for iFB+BA; Supporting Information: Figure S1) might alter the intracellular pH (J. H. Lee et al., 2019), thus affecting the activity of enzymes in the N‐glycosylation cascade in the Golgi apparatus (Chen & Harcum, 2006; Gawlitzek et al., 2000). Next to enzyme activity, BA might simply affect transcription rates of their respective genes, decrease their mRNA translation or lower the enzyme stability. It should also be considered that the increased *q*_*P*_ might cause a reduction in net glycan maturing time per IgG molecule as reported for a similar process strategy using N‐1 perfusion in combination with an iFB, including a lactate bolus feed to improve *q*_*P*_ (Stepper et al., 2020). Overall, the observed differences are small and the results are considered to be very comparable.

Regarding charged variants of the IgG (Figure 3g), no changes were detected between the processes. Directly after BA administration on Day 2.5, more acidic and less main and basic variants were found on Day 2.9. This does not have to be a consequence of BA supplementation but could also be related to intermediate impacts of the feed, for example, due to higher osmolality. After about Day 3.5, both processes showed a similar increase in acidic variants. Because the process iFB+BA was harvested 2 days earlier, this results in a slightly different final composition on the day of harvest with 2.5% versus 2% basic, 29.5% versus 21% main, and 68% versus 77% acidic variants. The general increase of acidic variants toward the process end complies with other studies about CHO cells expressing mAbs (Weng et al., 2020) and can be caused by a variety of occurring effects during process time reviewed by (Du et al., 2012). Concluding, these data show that neither the final N‐glycosylation profile nor charge variants prohibit the use of BA for intensification as IgG synthesis rates improve upon its supplementation and posttranslational modifications are not affected.

### 3.2 Transcriptomics

#### 3.2.1 DGE analysis

**Figure 4:**
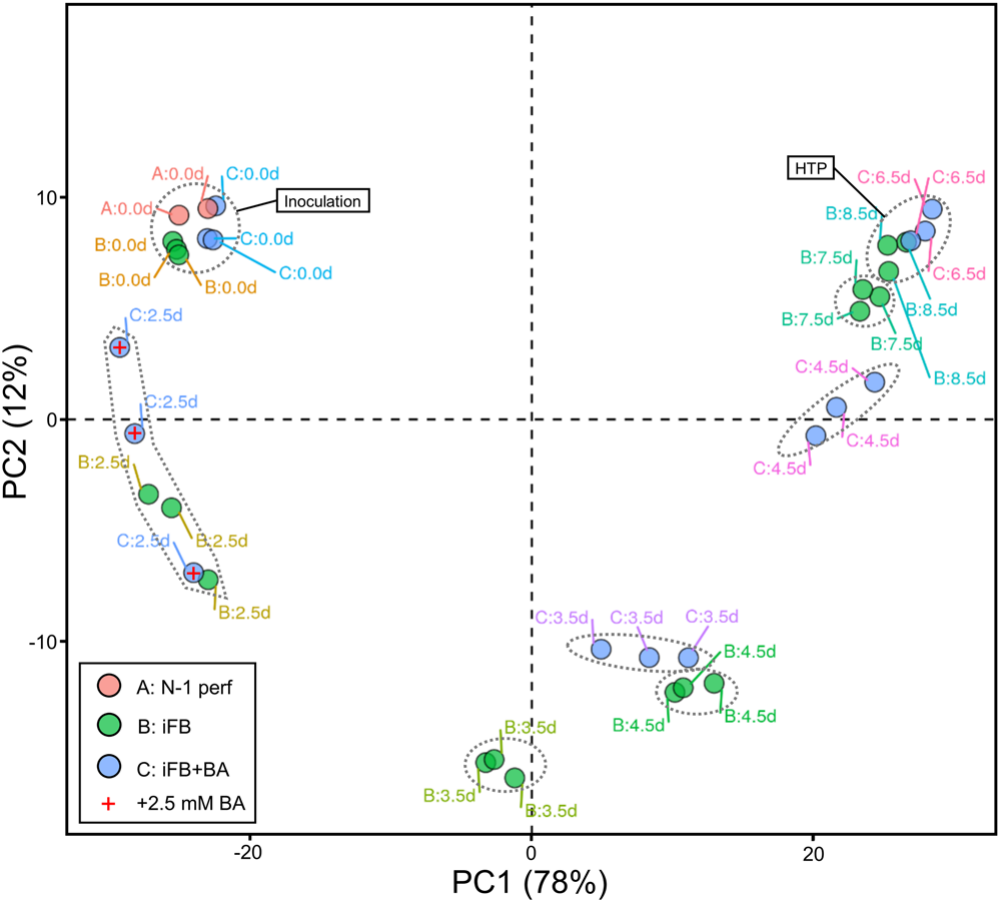
PCA plot of the transcriptomic results for the individual replicates of the different cell cultivations: Both N‐1 perfusion processes (Group A) were used to inoculate the iFB (Group B) and iFB+BA (Group C). Principal components 1 and 2 are shown. Dashed circles indicate clustered samples with respect to time. BA, butyric acid; HTP, harvest time point; iFB, intensified fed‐batch; PCA, principal component analysis.

We conducted a transcriptomic analysis to obtain mechanistic insight into the mode of action of BA supplementation in the iFB on the cellular phenotype observed, that is, reduced growth, accelerated viability decrease, and improved IgG production. A graphical overview of the variance is given by the principal component analysis (PCA) plot in Figure 4, where the first two principal components already cover 90% of the variance in the transcriptomic data (PC3 covers 7%; see Supporting Information: Figure S2). Furthermore, numbers of significantly differentially expressed (SDE) genes are given in Table 1. The PCA plot (Figure 4) shows that both the iFB (Group B) and iFB+BA (Group C) cluster with the gene expression state of their N‐1 perfusion process (Group A) directly before inoculation (day 6). Thus, potentially influencing factors, such as inoculation transfer time, change from rocked to stirred agitation, and media change (perfusion to production medium), did not have an impact on gene expression profiles confirming that both iFB cultures are suited for further transcriptomic analysis. Similarly, DGE analysis revealed no considerable differences in specific genes on the inoculation day (Table 1). On cultivation Day 2.5, that is, directly before BA supplementation, subtle differences in the cultures (267 vs. 366 SDE genes change as compared to Day 0) were observed for the iFB and iFB+BA, respectively. The root cause of this small variation might be the highly dynamic nature of the iFB, that is, they change from perfusion to fed‐batch as well as transition into the stationary phase at this time. The respective cluster appears stretched in the PCA plot along PC2 (Figure 4), but based on DGE analysis, both cultures are still very comparable (only 11 SDE genes; Table 1). At this time point, BA was supplemented such that more prominent differences in the gene expression profile were expected in the following days. On Days 3.5 and 4.5, the observations separated group‐wise and disclosed 465 SDE genes. Notably, BA supplementation seems to accelerate the development of the gene expression profile, that is, the data points for the iFB+BA on Day 3.5 are closer to those of the iFB on day 4.5 instead of iFB on Day 3.5. Similarly, those of iFB+BA on Day 4.5 are closer to the ones of iFB on Day 7.5 instead of iFB on Day 4.5. In the end, both processes converge to a more identical gene expression pattern. The final comparison of the gene expression state at the harvest time point (HTP) showed that observations clustered together again and only 73 genes remained significantly differentially expressed. This points toward either a time‐dependent mode of action of BA that might fade out after a certain time and/or an overlay of the emerging death phase. Related to this, supplying 1mM valeric acid continuously in a CHO cell perfusion process has been found to initially reduce cell growth and increase *q*_*P*_, but this effect diminished after about 7 days and cells recovered (Wolf et al., 2019). The authors suggested that this may be a directed evolutionary effect. Such recovery might not be expected in discontinuous processes naturally as multiple inhibiting substances accumulate in every fed‐batch. Thus, spiking a certain compound in a fed‐batch does not act as selective as in a perfusion process, still, this supports the assumption of SCFAs having a time‐limited rather than long‐lasting impact.

**Table 1:** Number of significantly differentially expressed genes for the comparisons of the N‐1 perfusion (A), iFB (B), and iFB+BA (C) processes

Comparison		# of SDE genes
with N‐1 perfusion	B d0 versus A	3
	C d0 versus A	1
iFB+BA versus iFB	d0	0
	d2.5	11
	d3.5	456
	d4.5	465
Harvest time point	d6.5 vs. d8.5	73
iFB	d0 → d2.5	366
	d2.5 → d3.5	668
	d3.5 → d4.5	139
iFB+BA	d0 → d2.5	267
	d2.5 → d3.5	1212
	d3.5 → d4.5	240

Abbreviations: BA, butyric acid; iFB, intensified fed‐batch.

**Figure 5:**
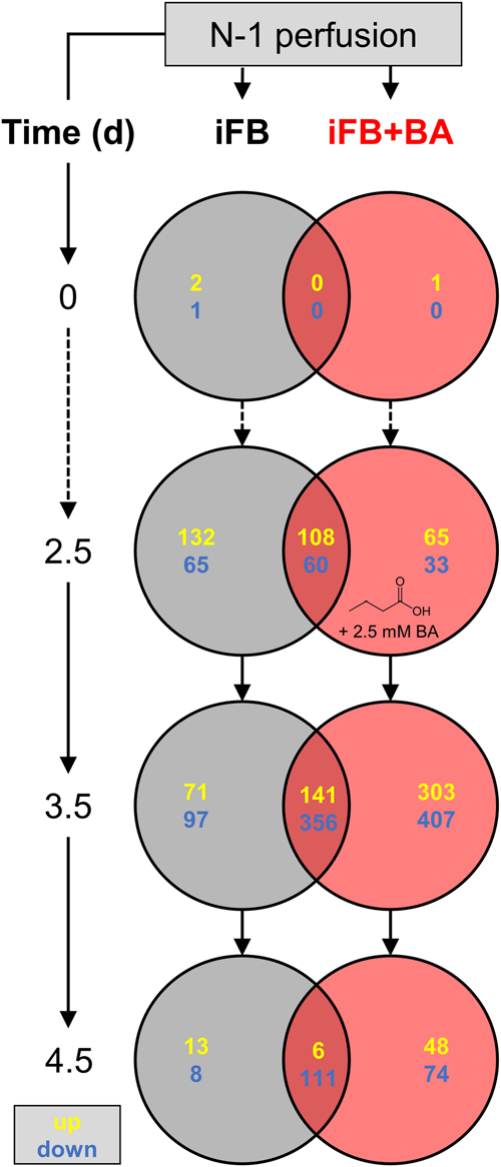
Venn Diagram of numbers of significantly differentially expressed genes for both processes, iFB and iFB+BA, from Day 0 until Day 4.5 compared with the previous day, respectively. Respective genes were then compared between both processes to identify specific and identical ones. BA, butyric acid; iFB, intensified fed‐batch.

To assess this aspect even further, the SDE genes were compared between both processes over time. The results are displayed in Venn diagrams in Figure 5. The figure shows SDE genes in the course of time for each process starting from inoculation on Day 0 until Day 4.5 and compares these genes at each time point with the gene expression of the previous day, respectively. Thus, up‐ or downregulated genes identical for both processes as well as specific ones can be quantified (the respective raw DGE results can be found in Supporting Information: File S1). Corresponding to previously described results, no differences were found on Day 0 and only small differences on Day 2.5. At this moment, BA was supplemented and one day later (3.5), a significant impact on the gene expression was discovered. The expression levels of 710 genes were uniquely changed due to this treatment, while only 168 were uniquely affected for the iFB. Roughly, 500 genes were common for both processes. In total, twice as many genes were affected due to BA supplementation. Generally, more genes were downregulated in all cases, which might reflect slowed down cell proliferation, as VCC peaked at this time point. Similar to these findings in the fed‐batch mode with 2.5 mM BA, a smaller concentration of 0.5 mM in a semiperfusion experiment using CHO cells to produce a RANK‐Fc fusion protein resulted also in distinctly more down‐ than upregulated genes (Fomina‐Yadlin et al., 2015). This illustrates that SCFAs tend to generally have a restraining holistic effect on gene expression patterns. One day later (4.5), almost no genes changed significantly for the iFB, indicating that the stationary phase was reached. For the process iFB+BA, more but still few genes were differentially expressed and it can be assumed that this is due to the sustained impact of BA. We conclude that BA affects gene expression, in particular, within the first 24 h after its supplementation.

**Figure 6:**
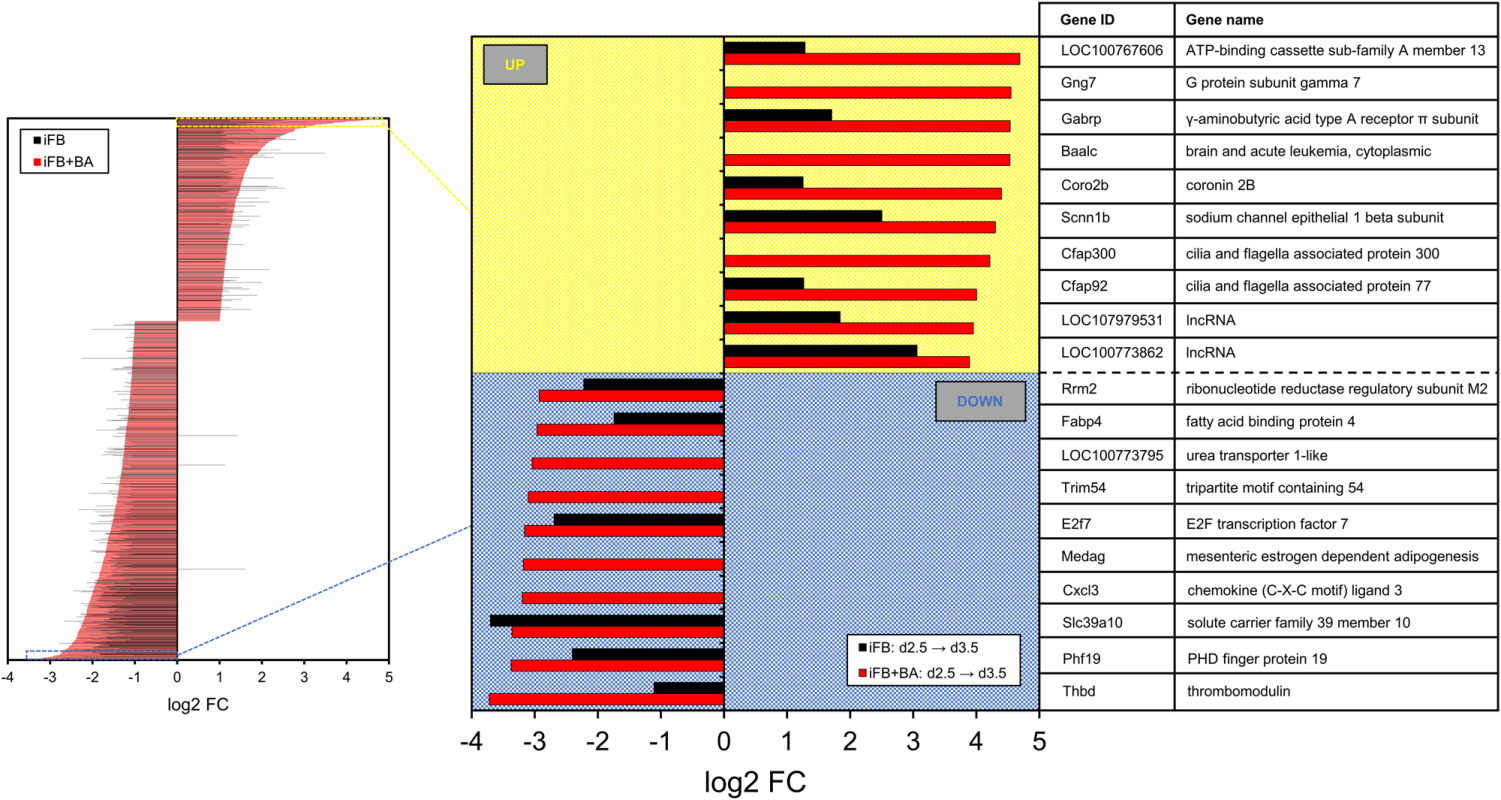
Top 10 significantly up‐ and downregulated genes from Day 2.5 to Day 3.5 sorted according to the log2 FC of the process iFB+BA. BA, butyric acid; FC, fold change; iFB, intensified fed‐batch.

To further elucidate genes affected by BA treatment from Day 2.5 to Day 3.5, the log2 FC of all 1207 SDE genes were considered and sorted from maximum (upregulation) to minimum (downregulation) in Figure 6. The respective 665 genes for the control (iFB) are also displayed. Only three of these genes are regulated in the opposite direction, that is, upregulated in the process iFB while iFB+BA shows downregulation. Two of them are long noncoding RNAs (*LOC103163365*, *LOC103163754*). The third one encodes for a matrix metallopeptidase 14 (*Mmp14*). In total, a high number of SDE genes (1061 of 1207) of the process iFB+BA showed higher absolute log2 FC values than the process iFB. This indicates that BA supplementation has no opposing effects and that it amplifies the up‐ and downregulation of expression of already differentially expressed genes compared to the process iFB. Among the top 10 upregulated genes of iFB+BA, three are unique being *Gng7*, *Baalc*, and *Cfap300* all of which cannot be assigned directly to any distinct biological function of CHO cells. The genes upregulated for both processes are all amplified for iFB+BA, for example, *LOC100767606* and *Gabrp*. Among the top 10 downregulated genes of iFB+BA, four are unique being *Cxcl3*, *Medag*, *Trim54*, which functions for CHO cells remain to be identified, and *LOC100773795*, which is a urea transporter. Again, almost all remaining ones are amplified for iFB+BA and mostly refer to cell cycle regulation. The transcription factor *E2f7* is related to the accumulation of cells in the G1 phase under ectopic expression (Di Stefano et al., 2003), and the ribonucleotide reductase subunit *Rrm2* limits DNA synthesis and repair (J. Li et al., 2018). Comparing these top 20 differentially expressed genes with the respective 139 genes of the process iFB from Day 3.5 to Day 4.5 (Supporting Information: File S1), only a few matched, and if so, with very low log2 FC. Thus, BA does not only act in a time‐accelerating manner. This initial DGE analysis showed that existing patterns in changes of the gene expression profiles are not only intensified due to BA treatment but also that it contributes to new genes becoming SDE. To further relate the DGE results to distinct and proper biological processes, molecular functions, or cellular compartments, GSEA was conducted using the normalized gene counts as input.

#### 3.2.2 GSEA analysis

**Figure 7:**
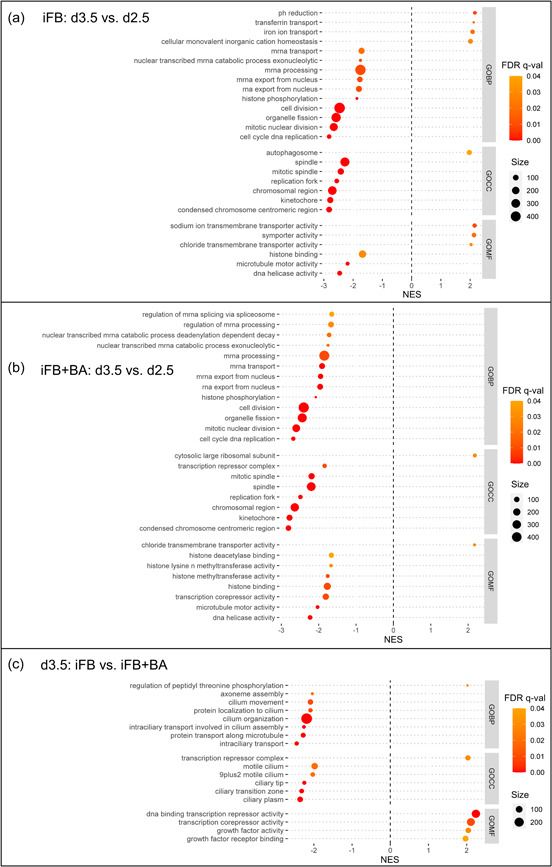
Dotplots of GSEA results on Day 3.5, that is, one day after BA supplementation, for the time series from Day 2.5 to Day 3.5 for the two processes (a) iFB and (b) iFB+BA as well as (c) their direct comparison on Day 3.5. Gene sets were sorted based on GO categories BP, CC, and MF. Gene sets with size >30, NES 1.5 < ≥ ‒1.5, and FDR *q*‐value ≤ 0.05 were considered as being significantly enriched from which distinct ones were selected to be displayed (see Supporting Information: File S2 for raw GSEA results). Positive NES indicates enrichment with the first state and negative NES with the second state of each comparison. BA, butyric acid; BP, biological processes; CC, cellular components; FDR, false discovery rate; GSEA, gene set enrichment analysis; GO, Gene Ontology; iFB, intensified fed‐batch; MF, molecular functions; NES, normalized enrichment score.

GSEA reveals whether a priori‐defined gene sets show concordant differences between two biological states (Subramanian et al., 2005). In this study, all three Gene Ontology (GO) categories biological processes (BP), cellular components (CC), and molecular functions (MF) were considered. Applying constraints (gene set size >30; NES 1.5 < ≥ ‒1.5; FDR *q*‐value ≤ 0.05) on the raw GSEA results (see Supporting Information: File S2) for each comparison, significantly enriched gene sets were identified. From these, biologically relevant sets were selected to be displayed in the dot plots (Figures 7 and 9) to either add value by explaining the observed phenotypes or present new hypotheses.

##### 3.2.2.1 One day after BA supplementation (Day 3.5)

Initially, the impact of BA one day after its supplementation was evaluated by comparing the biological states of both processes on Day 3.5 with Day 2.5 individually. This allows discriminating between gene sets being affected by BA supplementation and the ones that are not. The nature of every fed‐batch process, regardless of any potential modification, such as higher seeding concentrations or addition of productivity enhancers, is the transition from exponential to stationary growth phase, that is, cell growth slowing down. This can also be observed for both iFB (Figure 7a) and iFB+BA (Figure 7b) where identical gene sets with respect to cell proliferation were highly suppressed on Day 3.5 (NES < 0), for example, BPs, such as DNA replication, mitosis, and cell division. Accordingly, respective CCs, for example, the chromosomal region, kinetochore, or mitotic spindle, and MFs, for example, activities of DNA helicase and the microtubule motor were also found to be suppressed on Day 2.5. These observations were to be expected as they correspond with the transition from the exponential to the stationary phase at this time (Figure 2) when cell growth slowed down for both processes. Additional equally affected gene sets suppressed on Day 3.5 relate to histone binding and phosphorylation. The former implies reduced chromosome condensation and coiling, which interconnects with cell division. The effects of histone phosphorylation vary and depend on which histone (H2A, H2B, H3, or H4) and which residue is phosphorylated (comprehensive review, Rossetto et al., 2012). Here, it can be, for example, supposed that higher phosphorylation on serine 10 of histone H3 on Day 2.5 is associated with chromatin condensation related to mitosis (Wei et al., 1999). Yet, considering that further specific histone modifications were only enriched for iFB+BA on Day 2.5, this might also reveal effects on transcription regulation.

Effects on the transcription regulation can be assessed by sorting and classifying gene sets specifically enriched for iFB+BA. As such, suppressed histone deacetylase (HDAC) binding, which is in accordance with other studies designating BA as an HDAC inhibitor (Hong et al., 2014; Jiang & Sharfstein, 2008; Rahimi‐Zarchi et al., 2018), and histone methyltransferase activity were found to be enriched upon BA supplementation. Multiple phosphorylated histone residues were shown to be mechanistically linked to acetylation (Lau & Cheung, 2011) and demethylation (Metzger et al., 2010) of adjacent sites occurring in the same histone tail. The complex crosstalk between these modifications subsequently influences DNA condensation and packaging. Finally, they facilitate transcription by making gene copies more accessible to transcription factors and RNA polymerases (Jiang & Sharfstein, 2008), for example, the heavy and light chain (HC, LC) of the mAb.

**Figure 8:**
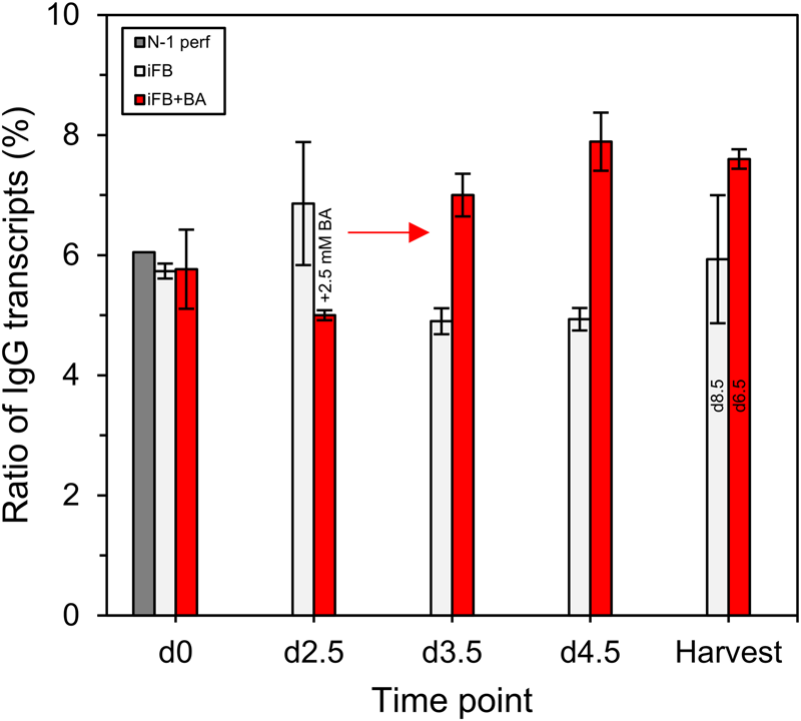
Ratio of IgG transcripts found in whole sequenced RNA. 2.5 mM BA was supplemented after 2.5 days. Harvest time point, that is, iFB after 8.5 and iFB+BA after 6.5 days. BA, butyric acid; iFB, intensified fed‐batch; IgG, immunoglobulin G.

In this study, facilitated transcription can be proved in two ways. First, gene sets related to its repression are enriched for the iFB on Day 3.5 compared to the iFB+BA (Figure 7c). Second, the fraction of mRNA transcripts of the IgG from total transcripts was increased by 2% to 3% following BA supplementation until the HTP compared to the untreated process iFB (Figure 8). The correlation of mRNA transcripts and cell‐specific productivity were shown to be linearly correlated and cell clone‐dependent (M. K. Jeon & Lee, 2007). In our study, transcripts correlate as well with the increase in *q*_*P*_ (Figure 2b). Still, to accomplish the translation of the mRNA transcripts into polypeptides, every cell utilizes further entities and functions of protein synthesis. These can also be derived from the GSEA results. After transcription, the pre‐mRNA is modified in the nucleus by 5ʹ‐capping, 3ʹ‐cleaving with poly(A)‐tailing and splicing. Regulation of the latter one was found to be enriched for iFB+BA on Day 2.5 (Figure 7b, NES < 0), which would impose enhanced splicing on Day 3.5 according to the rationale of increased protein synthesis. Afterward, the mature mRNA is exported from the nucleus to the cytoplasm, a process (Carmody & Wente, 2009) that was not affected by BA treatment as similar patterns were observed between the processes, that is, enrichment on Day 2.5. This poses the cellular capacities to accomplish the required export of an increased number of mRNA transcripts, eventually, as proliferating cells on Day 2.5 rely more on this mechanism. If an mRNA transcript was not properly processed, it would not be exported, but instead would be degraded by the nuclear exosome (Ogami et al., 2018). The respective gene set is enriched on Day 2.5 for both processes, thus it can be assumed that the increased transcription rates for iFB+BA do not result in a higher fraction of improperly processed transcripts. If the transport into the cytoplasm succeeds, translation is initiated by the ribosomes, of which the large subunit was enriched on Day 3.5 in the iFB+BA. This finding demonstrates increased protein production. The fate of every transcript certainly is cytoplasmic degradation, which starts via poly(A)‐trimming or its removal (Wiederhold & Passmore, 2010). The respective gene set, in turn, was enriched on Day 2.5 for the iFB+BA, which could recall for an extended cytoplasmic lifetime of transcripts after BA supplementation. Ultimately, distinct and central sections of protein synthesis were found to be enriched upon BA supplementation, which proves and explains the quantitative productivity improvement (Figure 3) that was also reported in other studies (Rahimi‐Zarchi et al., 2018; Yoon et al., 2004).

Besides the impacts on cell proliferation and protein synthesis, GSEA revealed further cell physiological characteristics to be influenced 1 day after BA supplementation that relates to intracellular homeostasis. For the control process (iFB), several gene sets were found to be enriched on Day 3.5 (NES > 0), for example, for iron transport via the glycoprotein transferrin. These gene sets were not significantly enriched for iFB+BA, thus indicating changes in the naturally occurring related biological processes. Intracellular proteins require iron as a cofactor for major biological processes. Its concentration is tightly regulated to cope with physiological demands to avoid excessive intracellular accumulation, which can catalyze the generation of reactive oxygen species (ROS), thus promoting oxidative stress (Dixon & Stockwell, 2014). Highly proliferating cells demand more iron for DNA replication (Chan et al., 1992; Laskey et al., 1988), and thus it appears natural that its transport eases on Day 3.5 in the iFB+BA as cell growth slows down more strongly. As this is not the case for iFB, the formation of transferrin and its receptor, both being vital for iron uptake, seems to be impacted by BA supplementation most likely as a consequence of cell growth arrest and facilitated transcription. Additionally, the iFB shows enrichment of sodium and chloride transmembrane transporter activity on Day 3.5, finally and naturally causing a pH reduction.

An important cellular component that was not found enriched for iFB+BA is the autophagosome, which is relevant for autophagy. Its lack of activation upon BA supplementation (J. S. Lee & Lee, 2012a) implicates that dysfunctional and degradable components remain and accumulate intracellularly, thus dooming cells to failure early on. Potential further evolution of the process iFB+BA would, therefore, make use of genetically or chemically controllable autophagy to profit from both advantages: BA (improved productivity) and autophagy (extended culture longevity) (Y. J. Kim et al., 2013).

##### 3.2.2.2 Two days after BA supplementation (Day 4.5)

**Figure 9:**
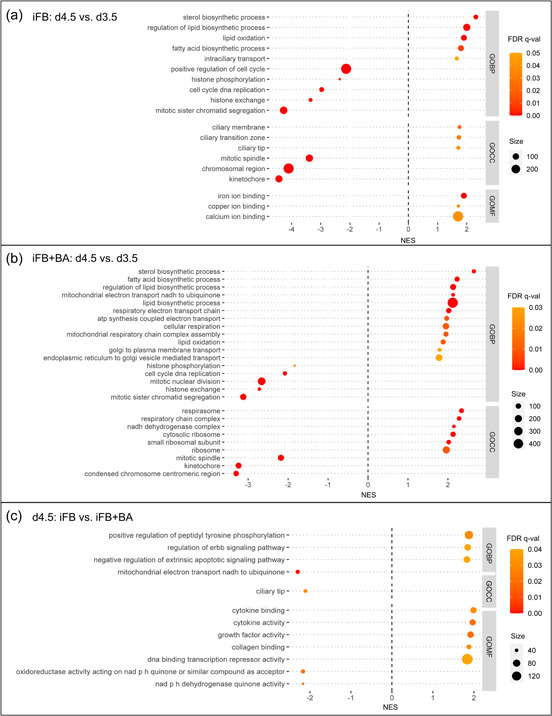
Dotplots of GSEA results on Day 4.5, that is, two days after BA supplementation, for the time series from Day 3.5 to Day 4.5 for the two processes (a) iFB and (b) iFB+BA as well as (c) their direct comparison on Day 4.5. Gene sets were sorted based on GO categories BP, CC, and MF. Gene sets with size >30, NES 1.5 < ≥ ‒1.5, and FDR *q*‐value ≤ 0.05 were considered as being significantly enriched from which distinct ones were selected to be displayed (see Supporting Information: File S2 for raw GSEA results). Positive NES indicates enrichment with the first state and negative NES with the second state of each comparison. BA, butyric acid; BP, biological processes; CC, cellular components; FDR, false discovery rate; GO, Gene Ontology; GSEA, gene set enrichment analysis; iFB, intensified fed‐batch; MF, molecular functions; NES, normalized enrichment score.

Following the time series from Day 3.5 to Day 4.5, the highest enrichment on Day 3.5 (NES < 0) was found for similar biological processes and cellular compounds as 1 day before, that is, related to cell proliferation for both processes iFB (Figure 9a) and iFB+BA (Figure 9b) further proofing cell growth reduction. Besides, both processes share enrichment of gene sets related to the biosynthesis of fatty acids and sterols on Day 4.5 (NES > 0), both being essential lipid constituents of cellular membranes. Considering the cell size increase starting after peak VCC on Day 3.5 (Figure 2) toward the process end, the regulation of lipid synthesis can be linked to the respective increases in biomass both quantitatively, that is, more biomass per cell, and qualitatively, that is, a higher lipid fraction in larger cells in the stationary phase, for example, by intracellular lipid accumulation (Pan et al., 2017). Thus, the remaining affected gene sets for iFB+BA can be traced back to the BA treatment. Important gene sets related to protein synthesis were identified, such as the ribosome and transport processes of intermediate proteins. As part of posttranslational modifications, vesicle‐mediated trafficking from the ER to the Golgi apparatus and from there to the plasma membrane for secretion of the mature protein was enriched. Different gene sets related to the respiratory chain were more strongly expressed on Day 4.5 for the BA‐supplemented process, which is also evident in the direct comparison of both processes at this time point (Figure 9c). Ultimately, this resulted in an enriched gene set on Day 4.5 relevant for ATP synthesis coupled to electron transport (Figure 9b). Respective reports for this phenomenon in mammalian biomanufacturing are scarce. A study investigating physiological changes in cell size during a CHO fed‐batch also found increased respiration and ATP synthesis with increased process time, that is, stationary phase, and respectively larger cells (Pan et al., 2017). More extensive reports, also considering the impact of BA, exist for related disciplines. Perfusion of isolated rat liver cells with butyrate revealed stimulation of oxygen consumption and an enhanced supply of reducing equivalents (NADH, FADH_2_) to the mitochondrial respiratory chain (Beauvieux et al., 2001; Gallis et al., 2007). The raised mitochondrial generation is then used to support ATP‐dependent gluconeogenesis (Nobes et al., 1990), which points to a deterioration of energy generation when exposed to BA for a certain duration. Transferring and applying these principles to biomanufacturing processes call for the investigation of techniques and strategies to prolong the adverse effects of BA supplementation described in this study.

One strategy could be the induction of autophagy, which can be realized by a targeted approach supplementing rapamycin during the process. Rapamycin is a specific inhibitor of the autophagy‐related enzyme mammalian target of rapamycin (mTOR) kinase (Zustiak et al., 2008), which eases the formation of autophagosomes (Chang et al., 2009), and was shown to inhibit CHO cell death (J. S. Lee & Lee, 2012b). In this study, the formation of the autophagosome (Figure 7a) was suppressed upon BA supplementation. The combined effect of rapamycin and BA has only been investigated on HeLa cells until now (Y. Jeon et al., 2007). Alternatively, it is worth investigating, whether additional genetic engineering of the cell line would allow the creation of a phenotype that is rather susceptible to apoptosis, which is one main physiological consequence of BA supplementation, for example, by transfecting cells with a vector of antisense RNA for caspase‐3 (N. S. Kim & Lee, 2002). By doing so, one could profit longer from BA's beneficial effects on protein production.

## 4 CONCLUSION

To cover the upcoming demands of biotherapeutics, biomanufacturing processes and principles need to be rethought. They can profit from technical progress by using typical intensification strategies, such as N‐1 perfusion for intensified fed‐batches. Further improvement can be attained by implementing techniques to increase cellular productivity, such as the addition of SCFAs, for example, BA. Combining these two principles, we established an intensified fed‐batch process supplemented with BA in bench‐top bioreactors. Our approach yielded improved IgG production capacities by maintaining higher cell‐specific productivity of over 25 pg c^−1^ day^−1^ whereas the control decreased below 10 pg c^−1^ day^−1^. Simultaneously, the N‐glycosylation and charge variant profile of the IgG as relevant product quality attributes remained unaffected. This proves the presented strategy a viable option to improve plant utilization on large scale. Furthermore, we subjected both processes to a holistic transcriptomic investigation using next‐generation sequencing with DGE and GSE analyses to understand the biological pathways underlying the observed experiment. Transcriptomic profiles of both processes are in line with an expected decrease in proliferation. Suppression of gene sets related to histone modification upon BA supplementation, that is, deacetylase inhibition and methyltransferase, complies with existing reports. We further identified checkpoints of protein biosynthesis, that is, transcription, mRNA processing and transport, ribosomal activity, and vesicular transport of IgG precursors in the cytoplasm, to be enhanced in the BA‐supplemented process, which ultimately explains the observed phenotype with increased productivity. Other biological processes affected include increased mitochondrial activity, the absence of autophagy stimulation, and suppressed the formation of transporter proteins for cofactors. Partially, these biological processes might serve as tackling points for the improvement and development of a cultivation strategy that outweighs or at least delays the detrimental effects of BA on cell viability, for example, by inducing autophagy and reducing apoptosis, thus extending culture longevity and with that potentially IgG yield. These strategies should be tested experimentally in the future, which could make such a process even more promising.

## AUTHOR CONTRIBUTIONS

*Conceptualization*: Markus Schulze, Julia Niemann, and Dirk E. Martens. *Experimental design and planning*: Markus Schulze, Julia Niemann, and Dirk E. Martens. *Execution*: Markus Schulze. *Data acquisition and analysis*: Markus Schulze, Yadhu Kumar, and Merle Rattay. *Writing and review of manuscript*: All authors.

## Supporting information

Supporting information.

Supporting information.

Supporting information.

## Data Availability

The data that support the findings of this study are available from the corresponding author upon reasonable request.
