# Supplementary material for: Transcriptomic analysis reveals mode of action of butyric acid supplementation in an intensified CHO cell fed‐batch process
Source: Biotechnol Bioeng. 2022 Jun 24;119(9):2359–73. doi: 10.1002/bit.28150 (PMC9545226; doi:10.1002/bit.28150)
Supplement: Supplementary file 1 — Supporting information. [file BIT-119-2359-s002.docx]

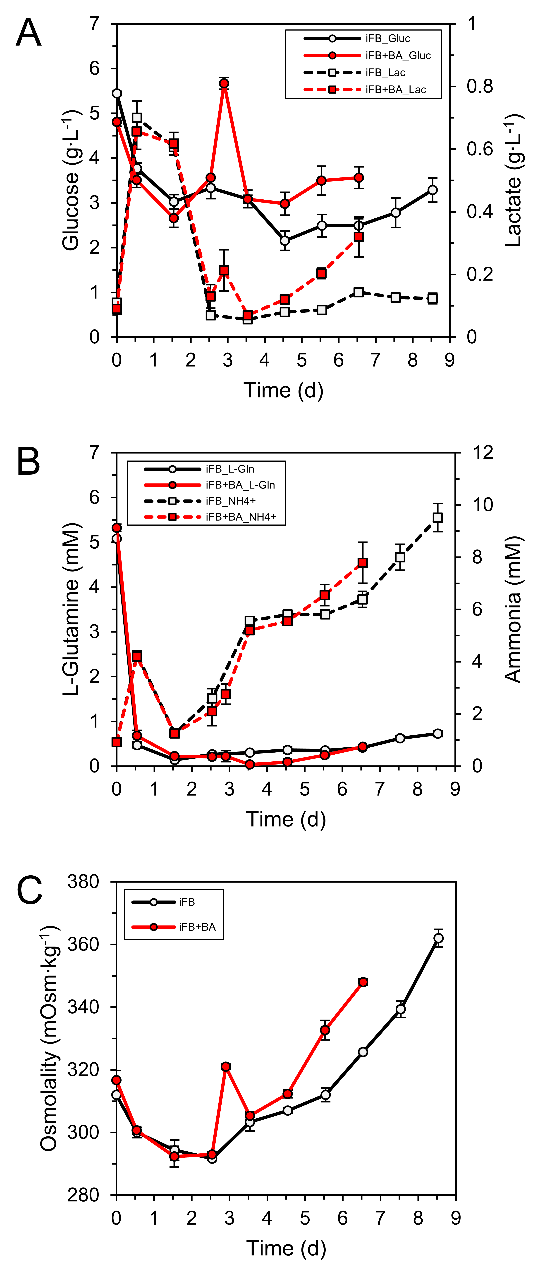


Figure S1: Profiles of A) Glucose concentraion, B) L-Glutamine concentration and C) osmolality over the course of process time. The control (iFB) was cultivated for 8.5 days, while the other process was supplemented with 2.5 mM BA at day 2.5 (iFB+BA) and cultivated for 6.5 days.


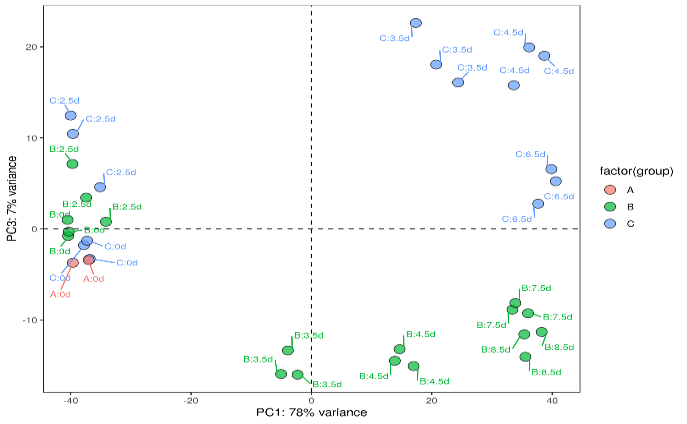


Figure S2: PCA plot of the transcriptomic results for the individual replicates of the different cell cultivations: Both N-1 perfusion processes (Group A) used to inoculate the iFB (Group B) and iFB+BA (Group C). Dashed circles indicate clustered samples with respect to time; HTP: harvest time point. Principal component 1 and 3 are shown.
